# TGF-β Signaling and the Epithelial-Mesenchymal Transition during Palatal Fusion

**DOI:** 10.3390/ijms19113638

**Published:** 2018-11-19

**Authors:** Akira Nakajima, Charles F. Shuler, Alexander O. D. Gulka, Jun-ichi Hanai

**Affiliations:** 1 Department of Orthodontics, Nihon University School of Dentistry, Chiyoda-ku, Tokyo 101-8310, Japan; 2 Department of Oral Biological and Medical Sciences, Faculty of Dentistry, University of British Columbia, Vancouver, BC V6T 1Z3, Canada; cshuler@dentistry.ubc.ca; 3 Massachusetts General Hospital, Center for Cancer Research, Charlestown, MA 02129-2020, USA; aogulka@mgh.harvard.edu; 4 Harvard Medical School, Department of Medicine, Boston, MA 02115, USA

**Keywords:** palatal fusion, cleft palate, TGF-β signaling, palatal medial edge epithelial (MEE) cells, midline epithelial seam (MES), epithelial-mesenchymal transition (EMT), collective epithelial migration, crowding

## Abstract

Signaling by transforming growth factor (TGF)-β plays an important role in development, including in palatogenesis. The dynamic morphological process of palatal fusion occurs to achieve separation of the nasal and oral cavities. Critically and specifically important in palatal fusion are the medial edge epithelial (MEE) cells, which are initially present at the palatal midline seam and over the course of the palate fusion process are lost from the seam, due to cell migration, epithelial-mesenchymal transition (EMT), and/or programed cell death. In order to define the role of TGF-β signaling during this process, several approaches have been utilized, including a small interfering RNA (siRNA) strategy targeting TGF-β receptors in an organ culture context, the use of genetically engineered mice, such as Wnt1-cre/R26R double transgenic mice, and a cell fate tracing through utilization of cell lineage markers. These approaches have permitted investigators to distinguish some specific traits of well-defined cell populations throughout the palatogenic events. In this paper, we summarize the current understanding on the role of TGF-β signaling, and specifically its association with MEE cell fate during palatal fusion. TGF-β is highly regulated both temporally and spatially, with TGF-β3 and Smad2 being the preferentially expressed signaling molecules in the critical cells of the fusion processes. Interestingly, the accessory receptor, TGF-β type 3 receptor, is also critical for palatal fusion, with evidence for its significance provided by Cre-lox systems and siRNA approaches. This suggests the high demand of ligand for this fine-tuned signaling process. We discuss the new insights in the fate of MEE cells in the midline epithelial seam (MES) during the palate fusion process, with a particular focus on the role of TGF-β signaling.

## 1. Introduction

**Figure 1:**
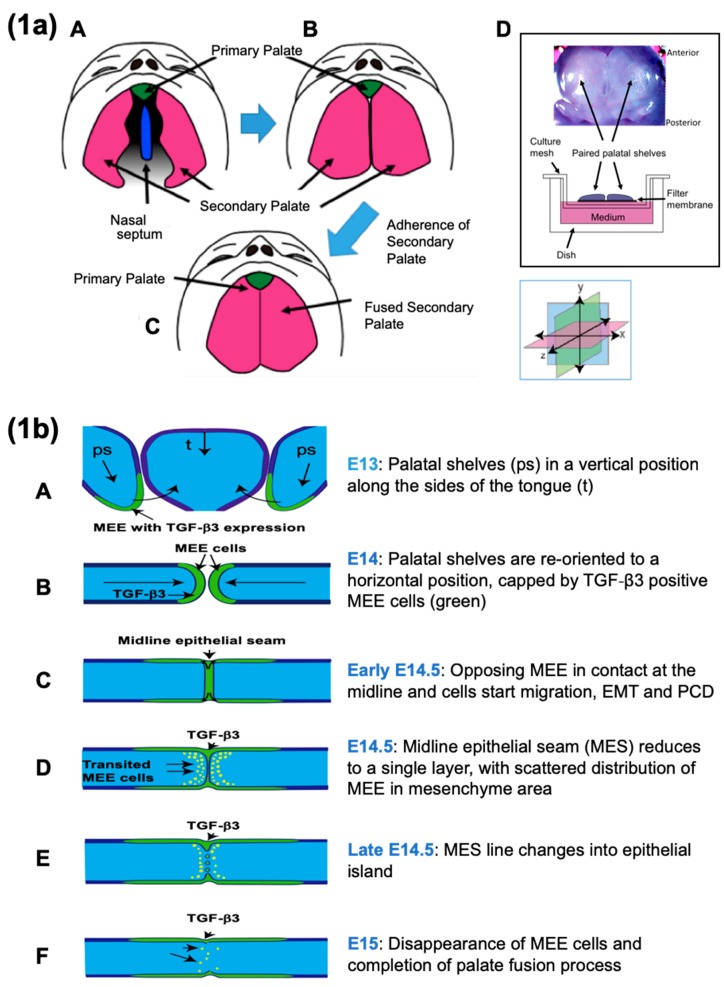

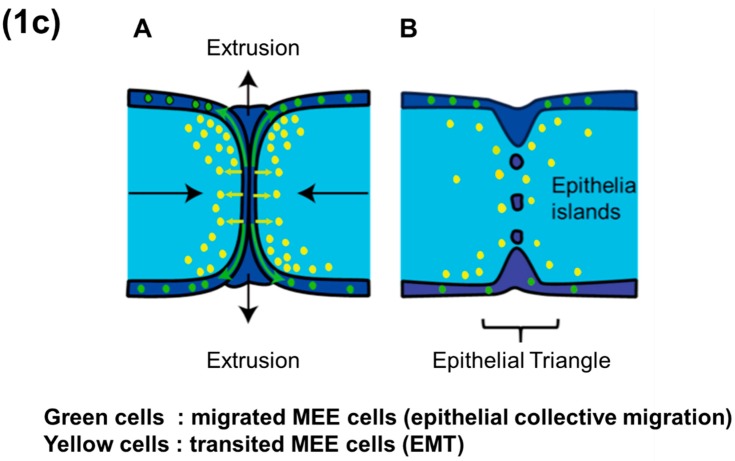
Intraoral outgrowths of palatal shelves and TGF-β3 expressions in medial edge epithelium (MEE) (schema). (**1a**) Development of the human secondary palate. (A) Early in palatogenesis, the shelves of the secondary palate grow vertically on either side of the tongue, with a gap between the secondary palate, nasal septum and primary palate. (B) After the descent of the tongue (not pictured), the palatal shelves elevate and re-orient horizontally (along with x axis; medial–lateral axes), allowing inter-shelf contact and the initiation of fusion. (C) By developmental week 12–13, palatal fusion is completed (Image modified from References [45,46]) (D) Actual and Schematic views of ex vivo experiment (tissue culture) using paired palatal shelves. (**1b**) Palatal fusion in mice. (A) Both palatal shelves grow vertically beside tongue at E13.0, and thereafter (B) both palatal shelves elevate and grow horizontally above the tongue at E14.0, and (C) continue to grow horizontally at the early stage of E14.5 coincident with strong expression of TGF-β3 [2,12]. (D) From this time, both epithelial-mesenchymal transition (EMT) processes and apoptotic changes are observed among the MEE cells at midline, and also cells of the MEE seam migrate collectively (as clustered aggregates; epithelial migration) into oral and nasal epithelial cell layers. At the middle stage of E14.5, the MEE seam changes from multiple layers to a single layer until they adhere in the midline seam [2,12]. (E) At the late stage of E14.5, EMT processes, epithelial migration and apoptotic changes still remain observable among the MEE at midline [10,11,12]. TGF-β3 expression is continuously strong during throughout E14.5 [2,12]. (F) Finally, the epithelium disappears from between the two apposed shelves, thus allowing complete palatal fusion by E15.0 [2]. (Dark blue—epithelial cells; light blue—mesenchymal cells; green—TGF-β3 expressions; ps—palatal shelf; t—tongue). (**1c**) Palatal fusion process after the contact of each palatal shelves (detailed schema, the main focus of this manuscript). (A) The MEE cells at midline epithelial seam (MES) cause epithelial cell (collective) migration, EMT, and apoptosis (which might be due to lethal EMT). Crowding force due to epithelial migration causes cell extrusion at the epithelial triangles, releasing MEE cells to the oral and nasal surfaces of the palate. (B) Because of these multiple biological events, the palate structure is reshaped and the MEE cell number at MES is decreased, causing epithelial island and disappearance of MEE cells finally. Images modified from Reference [46,47].

**Figure 2:**
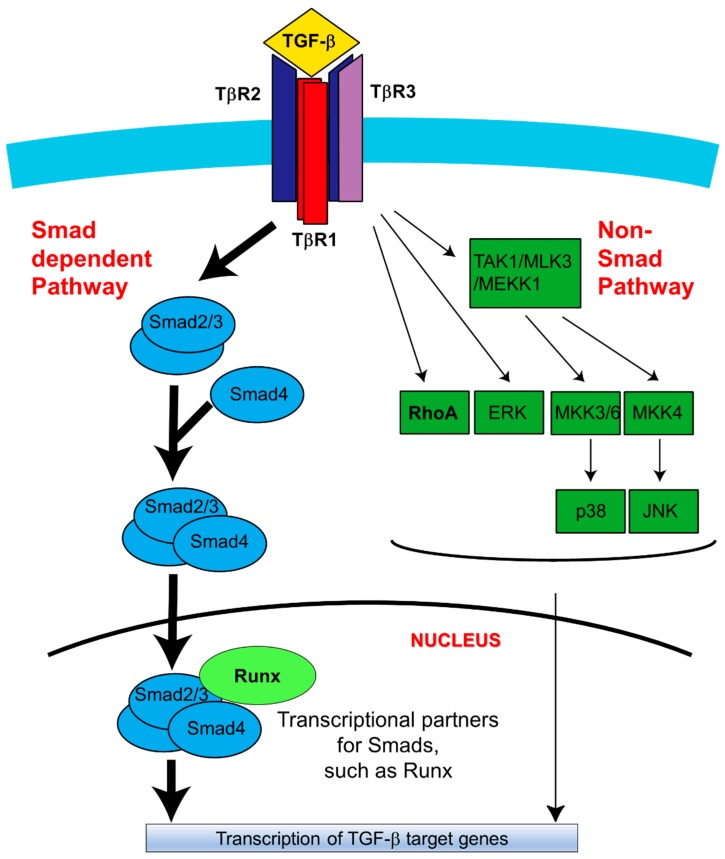
Schematic diagram of TGF-β signaling in palatal fusion. In the Smad-dependent signaling pathway, TGF-β initiates signaling by assembling receptor complexes that activate Smad transcription factors [25]. Initially, the ligand (TGF-β1, TGF-β2 or TGF-β3) induces downstream signaling by binding to TβR2 [25]. Upon the TβR2-TGF-β complex formation, TβR2 is phosphorylated and activated by TβR3 [34]. TβR1 is subsequently recruited to the complex and activated by TβR2-mediated phosphorylation in TβR1 GS region (a GSGS sequence) [34]. Through being bound by TGF-β ligands, TβR3 promotes complex formation and activates downstream signaling. Then receptor-associated Smads (Smad2/3; R-Smads) are specifically phosphorylated by TβR1 [34]. This phosphorylation induces dissociation of R-Smads from the receptor complex, thereby allowing them to associate with Smad4 and undergo translocation to the nucleus to mediate activation or repression of TGF-β target genes [25,34,48]. Alternatively, TGF-β can also activate non-Smad signaling pathways, including MAPK pathways (such as the ERK, TAK1, p38, and JNK), PI3K signaling, and RhoA-ROCK signaling [49]. Activation of these pathways has been identified under certain physiological and pathological conditions [34,48]. During the palatal fusion process, Smad2 is preferentially activated in the Smad-dependent pathway [34,48,49]. Among the non-Smad pathways, the TAK1-p38 axis is reported to activate Stat3 transcription to promote the EMT process [50]. PI3K signaling activation is also critical for palatal fusion as a downstream target of both Smad and non-Smad pathways [48,49]. Image modified from Reference [34]. TAK1: Transforming Growth Factor β -activated Kinase 1, MAPK: Mitogen-activated protein kinase, MLK3: Mixed-Lineage Kinase-3, MEKK1: Mitogen-activated protein kinase kinase kinase 1, MKK3/6: Mitogen-activated protein kinase kinase 3/6, ERK: extracellular signal–regulated kinase, JNK: c-Jun N-terminal kinases, PI3K: Phosphoinositide 3-kinase, RhoA: Ras Homology Family Member A, ROCK: Rho-associated protein kinase.

**Figure 3:**
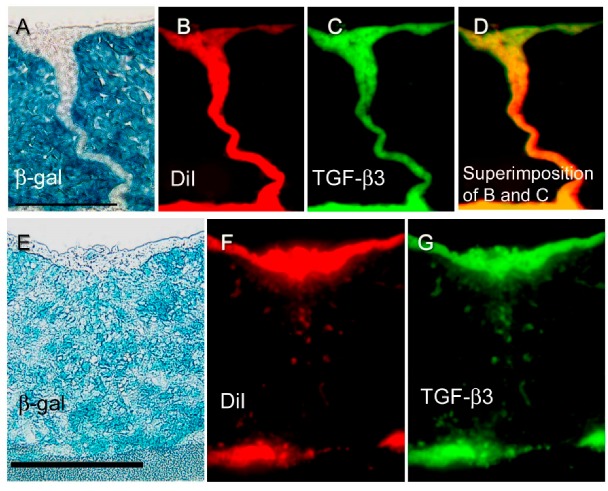

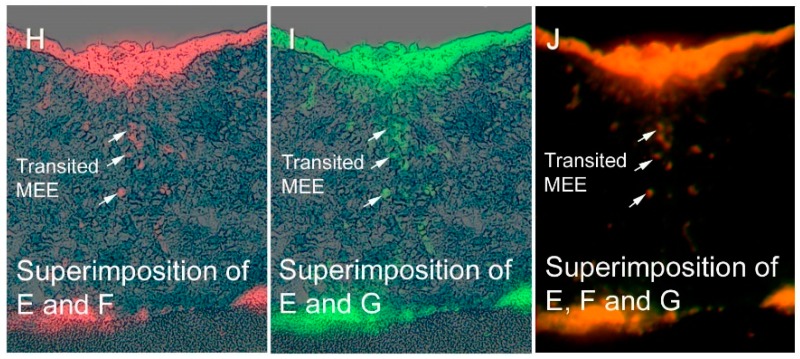
Distribution of β-gal, DiI, and TGF-β3 expression during palatal fusion. Expression and localization of β-gal, Dil, and TGF-β3 detected in vitro with immunofluorescence studies conducted in an organ culture at E13 + 24 h (**A**–**D**) and at E13 + 72 h (**E**–**J**). (**A**) At E13 + 24 h, the midline MEE cells were β-gal (−), and (**B**) DiI (+) and (**C**) TGF-β3 expression. Superimposition of DiI and TGF-β3 signals is shown in (**D**) in yellow. At E13 + 72 h, both palatal shelves were completely fused. (**E**) Most of the mesenchyme cells present are β-gal (+), although (**F**, **H**) some of the mesenchyme cells are β-gal (−)/DiI (+), or (**G**,**I**) β-gal (−)/TGF-β3 (+), at the midline seam. Superimposition of DiI and TGF-β3 signals is shown in (**J**) in yellow. Arrows indicate β-gal (−)/DiI (+)/TGF-β3 (+) cells that represent the transited MEE (**H**–**J**). These results demonstrated that transition MEE are strongly associated with TGF-β3 function in palatal fusion. Scale bars represent 50 μm in panels A and E. Images were obtained with permission from Reference [21] and modified, copyright Springer Press.

Cleft lip with or without cleft palate is one of the most common craniofacial birth defects in humans [1] and can occur as a consequence of defective secondary palatal fusion [1,2]. Fusion of the secondary palate takes place in the following three steps; (1) Two palatal shelves develop symmetrically on either side of the tongue, and grow vertically downward to a sufficient size to permit contact with between opposing shelves following rotation to a horizontal position (Figure 1a(A)), (2) At 6 weeks in human development the opposing palatal shelves reorient to a horizontal position above the tongue, such that the two palatal shelves come into contact (Figure 1a(B)), (3) the medial edge epithelium (MEE) at the palatal midline seam fuses and eventually disappears to complete the palatal fusion at around 12–13 weeks in human development (Figure 1a(C)) [2]. Thus, the MEE plays an important role in the fusion of the secondary palate during palate development [2]. Palatogenesis begins in mice at embryonic day 13 (E13), when the palatal shelves are positioned vertically beside the tongue (Figure 1b(A)) [2]. Thereafter, the tongue drops and both palatal shelves rotate to a horizontal position at E14 (Figure 1b(B)) [2]. During the early stage of E14.5, the palatal shelves meet at the midline and the opposing MEE adhere, resulting in the formation of a multi-layer epithelial seam (Figure 1b(C)) [2]. Later in E14.5, the palatal MEE seam changes to form a thin single cell layer (Figure 1b(D),c(A)). Following this, the MEE seam becomes discontinuous, leaving behind epithelial islands, while MEE cells accumulate at the oral and nasal aspects to form epithelial triangles (Figure 1b(E),c(B)) [2]. By E15, the MEE cells are no longer observable, and only mesenchymal cells are observed at the midline of the palate (Figure 1b(F)) [2].

The MEE cells have a key role in the completion of fusion, and as a consequence in mesenchymal continuity in the secondary palate. Three different fates may be adopted by MEE cells: (1) Programed cell death [3,4,5,6,7,8,9], (2) migration into the oral and nasal epithelia [8,10,11,12], or (3) epithelial-mesenchymal transition (EMT) [8,10,11,12,13,14,15,16,17,18,19,20,21,22]. The EMT process that occurs in MEE cells is induced by Transforming Growth Factor (TGF)-β3 and its receptors, which are highly expressed in MEE cells along the midline seam [20,21]. The presence of TGF-β3 is critical for palate fusion, as evidenced by the presentation of a completely cleft palate in TGF-β3 null mice, despite these mice having palatal shelves of sufficient length and exhibiting spatially and temporally appropriate shelf reorientation to permit contact and fusion [23,24].

Some of the MEE-derived mesenchymal cells are lost to apoptosis. Following the completion of palatal fusion, the remainder of the MEE-derived mesenchymal cells, characterized by their sustained high expression of TGF-β3 and TGF-β receptors, are adopted into the palatal mesenchyme. Thus, TGF-β signaling and TGF-β induced EMT processes are critical for the development of the palate, and more specifically for completing the process of palatal fusion [18,19,20,21,22].

TGF-β has three isoforms, including TGF-β1, -β2, and -β3 [25]. Among these, TGF-β3 is strongly expressed in the medial edge epithelium (MEE) (Figure 1a,b) [21]. All three TGF-β receptor isoforms are expressed in the MEE; two are serine/threonine receptor kinases, type I receptor (TβR1) and type II receptor (TβR2), and the third is an accessory receptor TGF-β type III receptor (TβR3, βglycan) [20,21,25,26,27,28,29,30,31,32,33,34,35]. Recently, TGF-β signaling has been shown to be regulated by both Smad-dependent and non-Smad dependent pathways (Figure 2). Additionally, multiple other molecules have been shown to play an important role in regulating developmental events, including bone morphogenetic proteins (BMPs) [36,37,38], FGFs [39,40,41], Wnt [7], Ephrins [42] and extra cellar matrix components [43,44]. Here, we present recent advances in TGF-β signaling research as they relate to the fate of the MEE cells during palate development.

## 2. The Role of the TGF-β Signaling Pathway in Palatal Fusion

### 2.1. MEE Cell Fate Includes Program Cells Death, Cell Migration and Epithelial-Mesenchyme Transition

Three types of cell fate have been characterized for MEE cells: Programed cell death (PCD) [3,4,5,6,7,8,9], cell migration [8,10,11,12], and EMT [8,10,11,12,13,14,15,16,17,18,19,20,21]. Accumulating evidence suggests that the EMT is strongly associated with TGF-β signaling during palatal fusion [8,10,11,12,13,14,15,16,17,18,19,20,21], and apoptosis may also be instigated by TGF-β signaling [34]. Using cell fate tracking approaches, it has been observed that MEE cells are present in the palatal mesenchymal region both during and after palate fusion [8], demonstrating the significance of both cell migration and the EMT in palate fusion [8].

A previous study examined derivatives of both cranial neural crest (CNC) and epithelial DiI cell lineages, using immunohistochemistry with the aim of characterizing MEE-related expression of TGF-β3 in in vitro palatal organ culture (Figure 1c) [21]. This study used cross breeding of Wnt1-Cre mice [51] with Rosa26 mice [52], generating Wnt1-Cre/R26R double transgenic mice with β-gal labeling of the CNC and all derivatives [53]. The epithelium of the palatal shelves was labeled with DiI for MEE cell lineage analysis [14,15,21]. The triple labeling (β-gal labeling/DiI labeling/TGF-β3 immunostaining) methods were performed on palatal tissue specimens isolated at defined stages of palatal fusion [21]. Interestingly, MEE-derived mesenchymal cells, which were characterized as β-gal (−)/DiI (+) cells, were positive for TGF-β3 expression in the fused palatal mesenchyme in this system (Figure 3A–D) [21]. When a fluorescent cell lineage tracer for the Cre-lox system was used to differentiate for CNC-derived or non-CNC mesenchyme cells, MEE-derived mesenchyme cells, which were identifiable as non-CNC/DiI positive/TGF-β3 positive mesenchymal cells, were observed in the mesenchyme area. The presence of these post-EMT MEE cells suggested that an EMT process occurred in the MEE during seam disintegration [21].

During EMT, a remodeling of the extracellular matrix (ECM) occurs and has an important role in signaling modulation; ECM remodeling is regulated by matrix metalloproteinases (MMPs), tissue inhibitors of metalloproteinases (TIMPs) and Periostin, affecting cell proliferation, migration, differentiation, and the EMT process [43,44]. Expression of MMPs has been examined in isolated transited-MEE cells by using a laser capture micro-dissection technique [21]. Using this method, it was also observed that *MMP13* mRNA was strongly and precisely expressed at the locus of contact between both palatal shelves and the transition-MEE cells [21,43]. This temporally and spatially coordinated expression of MMP13, together with the elevated expression of TGF-β3, might be critical for determining the fate of transiting MEE.

### 2.2. Epithelial Migration, Extrusion, and Apoptosis at the MES

The convergence and initial contact of palatal shelves is followed by a phase of MEE cell migration. In conjunction with this migration, crowding forces and associated stretch receptor activation-mediated signaling lead to epithelial cell extrusion and consequent devolution of the MES into distal epithelial triangles and medial epithelial islands. Ultimately this leads to the loss of all MEE cells, where the final stage of MEE cell disappearance, occurring at the completion of palatal fusion, might be a consequence of apoptosis (Figure 1a–c).

#### 2.2.1. Epithelial (MEE) Cell Migration

Live imaging of palate cultures has revealed that MEE cells move as a sheet-type aggregate rather than as individuals [54]. This collective migration process is regulated by Rho GTPase signaling, which may be activated via Smad and non-Smad signaling or by sphingosine-1-phosphate (S1P) signaling. This unique manner of migration has also been observed in epidermal wound healing, morphogenesis, vascular sprouting, and cancer invasion. Retention of intercellular contacts, coordination of actin dynamics between cells, and intracellular signaling [55,56] allows multiple cells form a structural and functional unit, which can then translocate across or through tissue [57] (Figure 1c).

#### 2.2.2. Extrusion

Epithelial cell extrusion, a process by which damaged or unwanted cells are expelled from the epithelium, can be instigated by the crowding signaling pathway or by apoptosis and is observed within the epithelial triangles during palatogenesis (Figure 1c(A)) [58]. Crowding activates the stretch-activated ion channel Piezo1, causing stimulation of S1P signaling, which in turn induces Rho GTPase-dependent extrusion [59]. Extruded MEE cells lose intercellular and cell-ECM connections, and in response undergo a form of programed cell death called anoikis. Apoptosis has been shown to directly activate S1P signaling, and thus can also contribute to the extrusion phenomenon.

#### 2.2.3. Apoptosis

**Figure 4:**
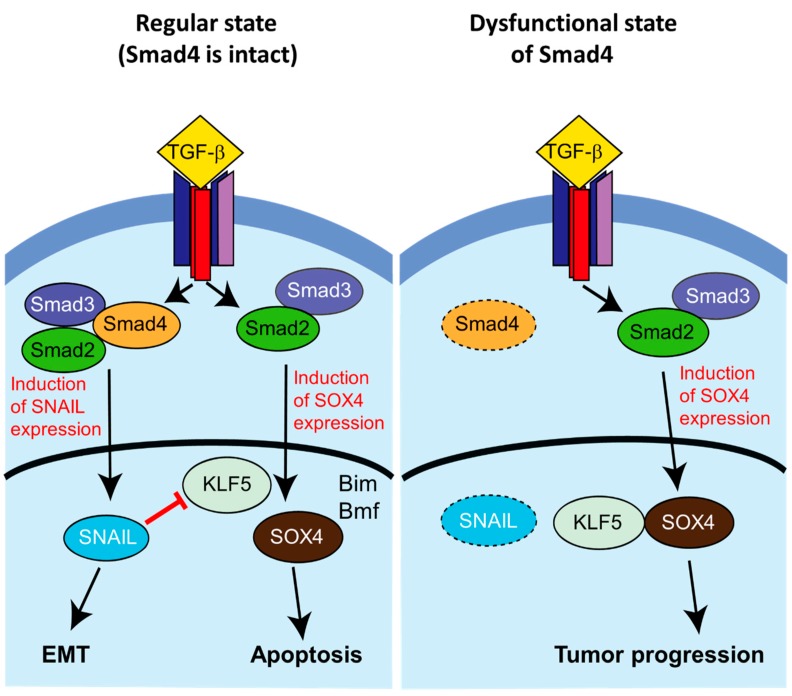
Smad4-dependent lethal EMT in a mouse model of pancreatic ductal adenocarcinoma (PDAC). (**Left**) In the presence of Smad4, stimulation by TGF- β causes EMT through activation of Snail and other EMT-related transcription factors via Smad2/3/4. *Snail* represses *KLF5*, another transcription factor. TGF-β also induces Sox4 expression via Smad2/3, which initiates post-EMT apoptosis (“lethal EMT”) through activation of transcription of *Bim* (*Bcl-2 interacting mediator of cell death*), *Bif* (*Bcl-2-modifying factor*), and other pro-apoptotic genes. (**Right**) In the case of loss of Smad4, TGF-β stimulation similarly causes induction of *Sox4* expression; however, in the absence of the Smad2/3/4 complex *Snial* and other EMT-related factors are not induced, and the de-repressed *KLF5* is able to act cooperatively with *Sox4* to promote the establishment of a pro-tumorigenic cellular state. Image modified from Reference [64].

Apoptosis has been suggested as an important player in the final stages of removal of MEE cells [6]. In models of Smad4 deficiency, MEE cell number in the MES is elevated and cells persist, avoiding elimination by apoptosis or other means as is seen during normal palatogenesis [60,61]. Smad4 has previously been implicated as an important player in cell proliferation versus death determination, notably in a model of pancreatic ductal adenocarcinoma (PDAC) [62]. In the absence of Smad4, it was shown that signaling along the TGF-β-Smad2/3 axis causes tumor cell proliferation through the cooperative activities of KLF5 and Sox4. In contrast, in Smad4-positive cells TGF-β-Smad2/3/4 signaling stimulates Snail expression, resulting in downregulation of KLF5 expression; without KLF5 modulation, Sox4 stimulation induces a “lethal EMT” response, wherein EMT is accompanied by apoptosis (Figure 4). In palate development, there is an evidence that many MEE cells remaining after fusion have weak Smad4 expression, suggesting that a similar lethal EMT response could be a possible mechanism for fate determination of MEE cells at the MES. KLF5 expression could be a key to confirming this hypothesis, which is supported by reports that Sox4 expression is highly restricted to the MEE cells at MES [63].

### 2.3. Human Syndromes with Palatal Defects Related to TGF-β Signaling

The incidence of human birth defects involving the lip and/or palate is reported to be 1.7 per 1000 births [1]. Non-syndromic cleft palate can be caused by exposure to various factors associated with genetic dysfunction, including certain drugs or toxins, as well as maternal smoking and/or alcohol consumption [65,66,67].

Mouse models and human genetic screens have implicated numerous genetic disorders in the aetiology of syndromic cleft palate, including dysostosis otomandibularis [68], Van der Woude syndrome [69,70], Smith-Lemli-Ovitz syndrome [71], Marfan syndrome [72,73,74,75,76,77,78], and Loeys–Dietz syndrome [79,80], and others. In particularly, Marfan syndrome and Loeys-Dietz syndrome involving the defect of the lip with or without palatal defect have been strongly associated with aspects of TGF-β signaling during palatogenesis [73,74,75,76,77,78]. Patients with Marfan syndrome exhibit craniofacial defects of the hard palate, as well as an abnormally tall stature with long limbs and long thin fingers, due to mutations in the *fibrillin-1* (*FBN1*) gene on chromosome 15 [73,74,75]. Previous evidence indicates that mutant FBN1 may directly bind to a latent form of TGF-β in the ECM, thereby sequestering and preventing its biological activity [78]. Loeys-Dietz syndrome exhibits a similar phenotype and mechanism as Marfan syndrome [79,80]. Importantly, there are five varieties of Loeys–Dietz syndromes [79], each of which are associated with mutations in TβR1, TβR2, Smad3, TGF-β2, and TGF-β3 respectively [79,80]. Among these, a heterozygous deletion of either TβR1 or TβR2 has been associated with craniofacial defects, including cleft palate [72].

### 2.4. Expression of TGF-βs in the Palate and the Resulting Phenotypes When Genes Related to TGF-βs Are Deleted

Around E13, TGF-β1 is expressed in both MEE cells and palatal mesenchyme cells in palatal shelves prior to fusion [81]. Expression of TGF-β1 then gradually decreases in palatal mesenchymal cells [81,82]. TGF-β1 null mice die 3 to 4 weeks after being born [83], and their immune functions, heart, and lungs are being most severely affected, however cleft palate has been reported in these mice [81,82,83,84] (Table 1).

TGF-β2 is also expressed in MEE cells and palatal mesenchymal cells when they adhere to opposing palatal shelves [85]. In TGF-β2-null mice EMT, cell growth, ECM production, and tissue remodeling are all adversely affected [85], leading to cardiac, lung, limb, spinal column, urogenital, eye, inner ear, and craniofacial defects [85] (Table 1). 

TGF-β3 is strongly expressed in MEE cells prior to the contact and fusion of opposing palatal shelves [19,20]. Thereafter, TGF-β3 continues to be strongly expressed during palatal fusion in the midline seam of palatal epithelial cells including EMT mesenchymal cells [19,20,21,23,24]. Interestingly, a complete cleft palate is observed in TGF-β3 null mice, even though the palatal mesenchymal shelves in this model have sufficient length and orientation to allow fusion [23,24]. Moreover, unlike other null mutants exhibiting a cleft palate, TGF-β3 null mice lack other concomitant craniofacial abnormalities [19,20,21,23,24,26,27,28]. Compared to other ligands, TGF-β3 is more specialized in its patterning of expression during palatogenesis and in its localization to the MEE and thus has the potential to fine-tune the fate of MEE cells toward migration, apoptosis or EMT [21,28,34] (Table 1).

### 2.5. Palatal Development and Expression of TGF-β Receptors (TβRs)

**Figure 5:**
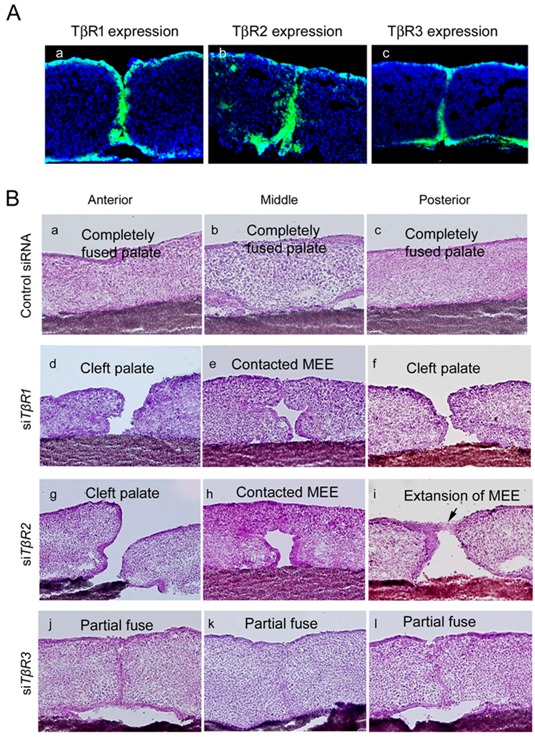
Expression of TβRs and the phenotype of palatal fusion at E13 + 72 h in a palatal organ culture after treatment with siRNAs targeting TβRs. Expression of TβRs was detected in cultured mouse palatal shelves in vitro. (**A**(a)) TβR1 expression was only detected in the palatal epithelium, (**A**(b)) TβR2 expression was observed in both the palatal epithelium and mesenchyme, and (**A**(c)) TβR3 expression was identified in the MEE cells. Representative phenotypes of the anterior region (**B**(a,d,g,j)), midline region (**B**(b,e,h,k)), and posterior region (**B**(c,f,i,l)) are shown. Palatal shelf organ cultures treated with a control siRNA (**B**(a–c)), siRNAs targeting *TβR1* (**B**(d–f)), *TβR2* (**B**(g–i)), and *TβR3* (**B**(j–l)) at E13 + 72 h are shown. Images were obtained with permission from Reference [35] and modified, copyright ELSEVIER Press.

There are three receptors in the TGF-β signaling pathway: TβR1, TβR2, and TβR3. TβR1 is expressed in palatal epithelial cells, including in the MEE (Figure 5A(a)). TβR1 null-mutant mice die at mid-gestation and exhibit severe defects in vascular development prior to bone formation [29]. TβR2 has the same expression profile as TβR1 (Figure 5A(b)), and homozygous TβR2 null mice exhibit defective yolk sac hematopoiesis and vasculogenesis [30,31]. As a result, embryonic lethality is observed around E10.5 [30,31].

In contrast with TβR1 and TβR2, TβR3 is strongly expressed in the MEE only during the palatal fusion stage [32,33,35] (Figure 5A(c)). TβR3 mutations in mice manifest in lethal proliferative defects in heart tissue and apoptosis in liver tissue at E13.5, indicating that TβR3 is required for somatic development in mice [86]. Interestingly, cardiac endothelial cells undergoing an EMT were also found to express TβR3 [86] (Table 1).

To identify the functional role of TβRs during palatal fusion, siRNA knockdown approaches have been utilized within a palate organ culture model [33,35]. In contrast to the control organ cultures wherein complete fusion of the anterior, middle, and posterior regions of the palate was achieved (Figure 5B(a–c)), *TβR1* knockdown organ cultures exhibited a cleft palate. This effect on palatal development appeared due to insufficient size of the palatal shelves and incomplete fusion was observed in the palatal shelves in the anterior and posterior regions (Figure 5B(d–f)) [33,35], and shelf contact at the middle region did not lead to fusion (Figure 5B(e)) [33,35]. Similarly, *TβR2* knockdown cases presented with completely cleft anterior palate (Figure 5B(g–i)), while the middle-palate failed to fuse despite shelf contact (Figure 5B(h)) [33,35]. *TβR3* knockdown showed a single layer of MEE cells remained in the midline of the anterior region of the palatal shelf junction (Figure 5B(j)), whereas in the midline of the middle and posterior regions this layer had devolved into MEE cells islands along the midline epithelial seam (Figure 5B(k,l)) [33,35]. This indicates the critical role of TβR3 and heterogeneous nature of its requirement over the course of palatogenesis, and collectively these results suggest that TβRs may contribute to spatial heterogeneity in the mechanism of MEE cell-fate regulation along the anterior–posterior and mediolateral axes in palatogenesis [87,88] (Table 1).

### 2.6. Smad-Dependent Signaling Pathway

Events downstream of the TGF-β signaling pathway include the assembly of receptor complexes, which primarily function to activate receptor-regulated Smads (R-Smads) [48,49,89,90,91,92,93,94,95]. Within the context of in TGF-β signaling, most are the R-Smads Smad2 and Smad3 [48,49,89,90,91,92,93,94,95] (Figure 2). Other developmentally relevant R-Smads include Smad-1, -5, -8, and -9, the downstream transducers in the bone morphogenetic protein (BMP) signaling pathway [48]. 

Initially, TGF-β isoforms bind to TβR2 [48], and this interaction recruits TβR1 to the TGF-β—TβR2 complex, causing TβR2-mediated phosphorylation of TβR1 at its glycine-serine (GS) rich region (a GSGS sequence) [48] (Figure 2, Left pathway). Smad2 and Smad3, which are recruited to the receptor complex through association with adaptor proteins [48], are subsequently phosphorylated at their carboxyl termini by TβR1 [48]. Following this, the interaction of these phosphorylated R-Smads with the co-mediator Smad (co-Smad) Smad4 facilitates nuclear translocation of the Smads complex and leads to the subsequent activation or repression of target gene transcription (Figure 2, Left pathway in the bottom) [36,48,94]. Smad4 is shared between the TGF-β signaling pathway and the BMP signaling pathway, and Smad4 makes specific contributions to each.

In the MEE, total levels of both Smad2 and Smad3 have been assayed, and interestingly only the phosphorylation of Smad2 has been observed in this context [25,48]. Notably, in the case of *Smad2* knockdown via siRNA, MEE cells were observed to persist at the palatal midline and the subsequent fusion process could not be promoted [95]. Moreover, the siRNA-mediated decrease in Smad2/phospho-Smad2 levels was also accompanied by an increase in the proliferation of cells in the MEE [92,94,95]. Thus, endogenous Smad2 expression appears to have a critical role in regulating the disappearance of the MEE (via migration, apoptosis and EMT) during palatal fusion [92,94,95].

### 2.7. Non-Smad Signaling Pathways

TβRs activate Smad-independent pathways that both regulate Smad signaling and induce Smad-independent TGF-β responses [49]. The latter activates mitogen-activated protein kinase (MAPK) pathways, including the ERK, JNK, and p38 MAPK kinase pathways [36,50,96,97,98,99,100]. Activation of the JNK and p38 MAPK pathways by TGF-β is also accompanied by TβR1 kinase activity-independent phosphorylation of TRAF6-TAK1 [50]. It has been suggested that p38 is strongly associated with palatal development in TGF-β3 null mice [100], and that this MAPK is activated by Tak1, a downstream transducer of TGF-β receptors complexes. In palatal fusion the TGF-β receptor -Tak1-p38 axis is requisite for the completion of the fusion process [99]. The TGF-β-induced EMT process is redundantly moderated by both Smad and non-Smad pathways during palatal fusion [100]. However, *Smad2* knockdown approaches resulted in inhibited EMT, suggesting that the non-Smad signaling pathway contributes less strongly to EMT than its Smad-dependent counterpart [95]. Precise functional roles for Smad-independent pathways during palatal fusion remain unclear. However, it is possible that Smad-independent pathways might affect the migratory or apoptotic character of MEE and/or the EMT process during palatogenesis (Figure 2, Right pathway).

## 3. Other Signaling Pathways and Possible Cross-Talks with TGF-β Signaling during Palatal Development

The previously reported factors that may affect palatal development are listed in Table 2. All signaling pathways, including these elements might play a role in proper completion of palatal fusion, with or without cooperative cross-talk with the TGF-β signaling pathway [101]. Here we introduce several signaling pathways, each of which has been well-established as significant within the palatal fusion context.

### 3.1. BMP Signaling

Expression of both BMP2 and BMP4, as well as their signaling target Msx1, has been detected in the palate [102]. BMPs are members of the TGF-β superfamily, and growth/differentiation signaling by BMP is one of the key regulators for palatal development [36,37,38,96,103,104]. Among other things, BMP signaling regulates cell proliferation, apoptosis, epithelial-mesenchymal interactions, and stem/progenitor differentiation during craniofacial development [37,103]. In the case of Msx1 deletion, BMP2/4 expression was reduced, and palatal cell proliferation was compromised in the anterior region of the secondary palate [104]. 

### 3.2. FGF Signaling

The fibroblast growth factor (FGF) signaling pathway is also critically involved in craniofacial development, and some roles for FGF have been identified in the development of the lip and palate [39,40,41]. For example, expression of FGF10 has been detected in the anterior palatal mesenchyme where it influences Sonic Hedgehog (SHH) expression, which in turn regulates BMP2 expression [41]. Meanwhile, FGF2 is expressed in the epithelium and in the mesenchyme of the middle and posterior regions of the palate [39,40,41], and FGF8 induces expression of Pax9 in the posterior region of the palatal mesenchyme [41]. 

FGF18 expression was detected in the mesenchyme during its change to a palatal MEE, and local application of endogenous FGF18 has been shown to induce ectopic expression of Runx1 in the epithelium of palatal explants [87]. In response to mesenchymal FGF18, Runx1 is also expressed in palatal shelf MEE cells [41,105]; moreover, Runx1 null mice exhibit partial clefting of the anterior palate, indicating the critical role of Runx1 in palatal fusion [41,105]. 

Notably, Runx1 is a binding partner of R-Smads in some contexts [25,41,105], and the Runx and Smad families share many biological functions [106,107]. Together with the reported significance of BMP signaling during palate fusion, the Runx-Smads association might be a key node, through which FGF, BMP and TGF-β signaling pathways converge to exhibit synergistic effects on MEE cells in midline epithelial seam (MES). 

### 3.3. Ephrin

The Ephrin (Eph) family of receptor tyrosine kinases and their membrane-bound ephrin ligands are responsible for many contact-mediated developmental processes, including multiple adhesion, migration, and boundary-forming events throughout development [42]. Binding of ephrins causes receptor activation in Eph-bearing cells (forward signaling), and intracellular signaling inside ephrin-bearing cells (reverse signaling) [42]. Ephrin-B reverse signaling in MEE cells is required for palate fusion, and this signal causes EMT in MEE cells through activation of Stat3 transcription, leading to the expression of EMT-related transcriptional factors Twist and Snail [42]. Stat3 may be a target of TGF-β signaling, while Ephrin-B reverse signaling and TGF-β signaling could function complementarily to activate Stat3 during the palatogenesis. Stat3 is reported to stimulate TGF-β3 expression in palate fusion [108] and has been shown to physically interact with Smad3 in order to activate EMT-related target genes in multiple biological contexts [109]. This suggests that there could be complementary mechanisms through which TGF-β signaling and Ephrin-B reverse signaling cooperate in order to promote EMT during palatal fusion.

### 3.4. Wnt Signaling

Canonical Wnt/β-catenin signaling plays an essential role in both development and diseases [7,42]. Several studies have implicated canonical Wnt/β-catenin signaling in the regulation of normal palate development [7,34]. β-catenin and several Wnt ligands and receptors are expressed in MEE cells, and epithelial-specific inactivation of β-catenin results in cleft palate formation and a reduction in GF-β3 expression, indicating that canonical Wnt/β-catenin signaling is a critical regulator of palate fusion through its role in maintain of TGF-β3 expression in MEE [42].

### 3.5. Extracellular Matrix (ECM) 

The ECM is an essential component of many biological processes, involving cell migration, proliferation, and differentiation [43,44]. The ECM also plays a critical role in mediating cell-cell interactions [43,110]. ECM turnover and properties are controlled by multiple enzymes [43,110], where MMPs, Tissue inhibitor of metalloproteinases (TIMPs) and Periostin, functioning at the cell surface or in the extracellular space, are particularly key [43,44,110]. During palatal fusion, as stated previously, the expressions of MMP2/13, TIMP2 and Periostin are highly induced in both MEE and transited-MEE, and MMP13 and Periostin are responsive to TGF-β3 stimulation around the midline seam [43,44]. Conversely, the epithelial cells responding to the pro-EMT growth factors, especially TGF-β, induce the neosynthesis of many ECM and cell surface proteins, causing remodeling of the local environment at the surface of transitioning cell [111]. Thus, TGF-β signaling is associated with ECM remodeling system, and synergistically enhances the EMT process during palatal fusion [43,44,110]. 

## 4. Conclusion and Perspectives

In this review, we discuss multiple strategies that have been used to define the MEE/mesenchymal cell populations that are present after palatal fusion and the association with the roles of TGF-β signaling. In particular, valuable insights have been gained from the isolation and analysis of β-gal/DiI cells [21]. Cell fate tracing approaches have unveiled the specific traits of populations of MEE-derived cells by using cell lineage specific markers, including a heritable marker for CNC cells, a cell lineage marker for the MEE, and molecules specific to the MEE during palatal fusion) [21]. 

TGF-β3 plays a dominant role in palatogenesis [23,24], and its fine-tuned expression is temporally and spatially correlated with the critical events surrounding palatal shelf adhesion [21]. For example, in TGF-β3 null mutant mice, the palatal shelves fail to adhere properly, the basement membrane is not degraded, and the MEE does not undergo EMT [23,24]. The high expression of TGF-β3 in MEE cells throughout during the palatal fusion process suggests a critical role of TGF-β3 for the fate of these cells during palatal development [8,10,11,12,13,14,15,16,17,18,19,20,21]. As highlighted in this review, the characterization of MEE-derived cells by cell fate tracking will allow us to elucidate their developmental fate following palatal fusion and unveil the contributions of multiple mechanisms, including the TGF-β signaling pathway.

Focusing on MEE cells undergoing cell migration/PCD/EMT during palatal fusion, we summarized the roles of the TGF-β signaling pathway. The population of the MEE-derived mesenchymal cells in the palatal mesenchyme is a consequence of EMT, primarily moderated by TGF-β3 [19,20,21,22,23,24,26,27,28]. TGF-β signaling may also be associated with the initiation of programed cell death [9]. Cell fate tracing for identification of specific traits will allow us possible to dissect the complicated developmental process in palatogenesis. In the meantime, there are further questions that need to be addressed. In particular:

1. What is the fate of MEE cells after they undergo EMT?

During embryonic development at the neural crest, EMT may be also followed by a mesenchymal-epithelial transition (MET) [100], where the mechanisms involved remain unknown. This combination of EMT-MET events is referred to as the EMT-MET cassette hypothesis. These sequential events may be relevant in cancer metastasis to explain tumor colonization after an EMT. For example, during the development of kidney tubules, mesenchymal cells undergo a MET via epithelization of the surrounding mesenchymal cells that are attached to the tubular epithelial cells [100]. However, the palatal fusion process and the role of MEE cells in this process after an EMT remain to be characterized.

2. What is the role of cross-talk between signaling pathways and how is EMT regulated? 

In several biological events related to the fate of MEE, the EMT process is enhanced by proteins such as BMP2/4, EGF, FGF, Ephrin, Wnt, Msx1, Runx1 and MMPs [36,37,38,39,40,41,42,43,87,88,103,104,105,106,107,108,109,110,112,113]. In addition, it is possible that the observed EMT during palatal fusion continues after palatal fusion has been completed. By gaining a better understanding and an insight into the biological processes involved and by possible interventional approaches, regulatory mechanisms pertaining to the EMT process may be more extensively characterized.

3. Regulation of TGF-β signal intensity during the palatal fusion process

The strikingly high expression of TGF-β3 and TβR1/R2/R3 in MEE at the midline seam, and their immediate downregulation after the process indicate the strength and sensitivity of regulation of their signaling intensity. The mechanisms underlying this signaling modulation are still unclear, but the presence of highly expressed TβR3 might be a key for this, and moreover the MMP activity of microenvironment might also play a role though the conversion of the TGF-β ligand from its inactive to active form [18,34,43,93]. Cross-talk with other signaling pathways is also a candidate mechanism for explaining TGF-β signaling dynamics [37,39,40,41,49,70,88,93,96,97,104,105,107,108,110,114]. Some further interventional approaches may unveil these underlying mechanisms.

## Figures and Tables

**Table 1:** The expression patterns and phenotypes of null mutants of TGF-β ligands and receptors prior to fusion.

	Localization	Phenotype at Null Mutant
TGF-β1	MEE (prior to fuse) and Mesenchyme	(-)
TGF-β2	MEE (prior to fuse) and Mesenchyme	23% (+)
TGF-β3	MEE	(+)
TβR1	MEE	? (Die at E10.5)
TβR2	MEE	(+)
TβR3	MEE	(-)

+: Cleft palate, -: Normal.

**Table 2:** The list of gene expressions associated with palatal fusion (modified from Yu et al. [101]).

Fusion Stage	Vertical Growth		Elevation (Before Fuse)		Adhesion (Contact and Fusion)		After Fusion (MEE Disappear)	
Cells	Mesenchyme	Epithelium	Mesenchyme	Epithelium	Mesenchyme	Epithelium	Mesenchyme	Epithelium
Ligand	*EphB2/B3*	*pERK*	*TGF* *β1/2*	*TGF* *β1/2*	*BMP2/3/4*	*TGF* *β3*	*BMP2/3/4*	
	*FGF7/10*	*pMEK*	*EphB2/B3*	*pERK*	*EphB2/B3*	*BMP3*	*Osr2*	
	*Wnt5a*	*Shh*	*FGF10*	*pMEK*	*FGF2/8/10/18*	*FGF2/18*		
			*FGFr1/2b*	*Shh*		*Shh*		
				*Wnt11*		*Wnt11*		
						*Smad2*		
						*pMEK*		
Receptor		*FGFr2*		*FGFr1*		*T* *βR1/2/3*		
				*FGFr2b*		*FGFr2*		
Transcriptional	*Snail*	*TBX1*	*Msx1*	*TBX1*	*Msx1*	*Snail*	*Snail*	
Factor	*TBX22*	*TBX22*	*Twist*		*Snail*	*TBX1*		
	*Twist*		*Snail*		*Twist*	*Twist*		
	*Msx1*					*Runx1*		
	*Pax9*							
Extracellular					*MMP2/13*	*MT-MMP*	*MMP2*	
Matrix						*MMP13*		
						*TIMP2*		
						*Periostin*		
